# Bridging gaps in age estimation: a cross-sectional comparative study of skeletal maturation using Fishman method and dental development using Nolla method among Egyptians

**DOI:** 10.1007/s00414-024-03394-x

**Published:** 2025-01-06

**Authors:** Heba Ibrahim Lashin, Asmaa Fady Sharif, Mohamed Salah Ghaly, Shaimaa Shaban El-Desouky, Amira Elsayed Elhawary

**Affiliations:** 1 Forensic Medicine and Clinical Toxicology Department, Faculty of Medicine, Tanta University, 6th floor, Medical Campus, El-Geish Street, Tanta, Gharbia 31527 Egypt; 2 Department of Clinical Medical Sciences, College of Medicine, Dar Al Uloom University, Riyadh, Kingdom of Saudi Arabia; 3 Pediatric Dentistry, Oral Health and Preventive Dentistry Department, Faculty of Dentistry, Tanta University, Tanta, Egypt

**Keywords:** Forensic odontology, Dental age estimation, Skeletal age estimation, Nolla method, Fishman method, Egyptians

## Abstract

Estimating the chronological age of humans is a prevalent need in forensic practice. Comparing the accuracy of different age identification methods helps provide the most reliable method for a specific population. This study aimed to compare the accuracy of age assessments using skeletal maturation and dental mineralization in a sample of Egyptians and to assess if combining both methods yields more precise age prediction. A cross-sectional study included 176 Egyptian children and adolescents aged between 8 and 16 who underwent orthopantomograms and hand-wrist radiographs. All radiographs were scored independently for skeletal maturation using the Fishman method and dental development using the Nolla method. While Fishman and Nolla methods were valid and reliable age predictors with variable sex and age group accuracy in Egyptians, the Nolla method showed superior performance. The Nolla method slightly underestimated the chronological age, while the Fishman method slightly over-estimated it, showing median differences of -0.21 and 0.17, respectively. Correlating the estimated age using the Fishman and Nolla methods and the chronological age showed intraclass correlation coefficients of (0.854 and 0.660) and (0.973 and 0.977) for females and males, respectively (*P* < 0.001). The model adopting the Nolla score exhibited the highest R² (0.973 and 0.968) and the lowest Akaike information criteria (96 and 106) for females and males, respectively, which is comparable to the model adopting both Nolla scores and skeletal maturation indicators. Therefore, we recommend adopting the model incorporating only the Nolla scores for age estimation without the need for further hand and wrist radiography.

**Supplementary Information:**

The online version contains supplementary material available at 10.1007/s00414-024-03394-x.

## Introduction

Age estimation is essential for understanding the growth and developmental changes in children. For such importance, chronological ages are documented in birth proofs [1]. Forensic age identification is one of the most important medico-legal factors in clinical practice and the court of law [2]. Estimating the chronological age of individuals or skeletal remains is a prevalent need in modern forensic practice and bioarcheology. Moreover, identifying children and young adults’ actual ages is one of the main challenges forensic pathologist faces [3].

Globally, age identification became more important with the dramatic increase in cross-border migration [4]. Many countries have reported increasing numbers of foreigners who cannot provide documentary evidence of their date of birth [5]. Moreover, age estimation is required, especially among children and adolescents, for civil purposes, such as adoption processes or criminal reasons, including minor prostitution and age fraud in several sports, particularly in those aged below 18 years [6]. Furthermore, to check whether the accused has reached a legal majority [7, 8]. Besides in mass disasters, an accurate age identification is helpful to narrow the search for possible victims and it has a crucial role in reconstructing the biological profile of unidentified dead bodies and skeletal remains [9, 10]. Besides its application in pediatric dentistry and orthodontics [11], age estimation is also critical in clinical practices, including managing different endocrinal and non-endocrinal disorders [12, 13].

Medical approaches based on assessing the bone maturation and dental mineralization are strongly related to chronological age in young adults, and for this reason, they can be used to estimate age [14]. The skeletal age is considered one of the most reliable methods for assessing chronological age, although it is affected by ethnicity [15]. The International Study Group on Forensic Age Diagnostics (ADFAD) recommended age prediction depending on skeletal maturation shown in hand and wrist radiography [2]. The wide use of indicators of skeletal maturation is owed to multiple factors, including simple techniques, minimal radiation exposure, and economic costs [16].

Skeletal age identification could be done using X-ray filming of different body parts to assess the degree of bone maturation. One of the most popular and easy methods used in pediatrics is X-ray filming of hand and wrist [13]. This method was described by Fishman [17] and seems to be the most appropriate method for assessing skeletal maturation. It assesses eleven skeletal maturity indicators (SMIs), covering the entire development period [17, 18].

However, human skeletal development could be influenced by nutritional and environmental factors. Hence, estimating the dental age is considered vital, as the teeth are less affected by genetic, hormonal, nutritional, and pathological changes compared to the bones [19]. Multiple methods have been studied for dental age estimation, However, there is no standard method that could be reliable for all populations [20]. This could be attributed to the fact that age estimations are generally population-specific [21].

Dental age estimation in children can be based on clinical tooth emergence or on the stages of tooth formation observed in radiographs, which is considered a better method to determine the chronological age than the tooth emergence as local and systemic factors scarcely influence it [22]. One of these methods is the Nolla method which is commonly used in teaching and clinical practice. It presents a high degree of intra-observer agreement (greater than 90%). Moreover, the results obtained by the Nolla method are no less reliable than those obtained by other methods [11, 23]. Despite its effectiveness, this method has been one of the least tested across different populations [11].

Comparing the accuracy of different age identification methods helps provide the most reliable and suitable method for a specific population or ethnic group [24]. Therefore, the relationship between skeletal maturation stages and tooth calcification by comparing different methods in different populations has been previously evaluated by multiple studies [16, 25–27]. However, to the best of our knowledge, no studies have been reported on the relationship between skeletal age assessment using the Fishman method and dental age assessment using Nolla method in Egyptian children, even though there may be racial differences in these children that would need more investigation. Therefore, this study aimed to compare the accuracy of skeletal age assessment using the Fishman method and dental age assessment using the Nolla method in estimating the chronological age in a sample of Egyptian children and adolescents. In addition, this study aimed to assess the validity of combining both methods for age prediction.

## Subjects and methods

### Sampling and sample size calculation

A convenience random sampling technique was adopted where the sample size was calculated using MedCalc Statistical Software version 19 [28]. The calculation assumed that the Bland-Altman plot was required to test the agreement between chronological age and the assessed methods, adopting an alpha level of 0.05 and a power of 80%. For dental age, an expected mean difference of 0.49 with a standard deviation of 1.02 and a maximum allowed difference of 3 [29] yielded a minimal sample size of 96 children. For skeletal age assessment, a mean difference of 0.23, a standard deviation of 1.19, and a maximum allowed difference of 3 [30] yielded a minimal sample size of 174 children. As eight age groups were included, the sample was rounded to 176, so that each group contained 22 children (11 females and 11 males).

### Study design and population

This prospective cross-sectional study was conducted on randomly selected 176 Egyptian children and adolescents (88 females and 88 males) aged between 8 and 16 years as a part of their clinical work up at the Outpatient Clinics of Pediatric Dentistry and Orthodontic Departments, Faculty of Dentistry, Tanta University during the period from the start of April 2023 to the end of March 2024. The included subjects were in need for panoramic and hand-wrist radiographs in their dental treatment. According to Alqadi and Abuaffan, the first SMI (1) appears around 9 years using the Fishman method. Hence, the studied sample was limited to children above eight years of age [30]. Children who did not reach SMI (1) were excluded from statistical comparisons since they could not be scored by the Fishman method [31]. Similarly, according to previous studies, the last SMI (11) appears around the age of 16.5. Hence, the studied sample was limited to children below 16 years of age to avoid the under estimation of skeletal age in adolescents aged more than 16 years [32, 33].

Both male and female samples were divided into eight groups, according to chronological age as follows: Group 1 (8-8.99 years), Group 2 (9-9.99 years), Group 3 (10-10.99 years), Group 4 (11-11.99 years), Group 5 (12-12.99 years), Group 6 (13-13.99 years), Group 7 (14-14.99 years), and Group 8 (15-15.99 years).

### Eligibility criteria

Egyptian children of normal development (50th percentile growth charts) aged between 8 and 16 years presented to the Dental Outpatient Clinics, Faculty of Dentistry, Tanta University for teeth development assessment or orthodontic treatment with no agenesis or extractions in the left lower quadrant in the selected panoramic X-rays were included in this study. Children with premature birth, facial asymmetry, congenital anomalies, bone diseases, or systemic diseases affecting the growth and development of teeth were excluded from the study. Also, children with a history of trauma or surgery in the dentofacial region and malignancies or previous chemotherapy or ionizing radiation treatment were excluded. In addition, patients with previous or current orthodontic treatment or poor-quality radiographs or who were unaware of their date of birth were not included in the study.

### Data collection method

The sex of the studied individuals was determined using birth proof. The chronological age was calculated by subtracting the date of radiographic examinations from the date of birth from the after converting both to a decimal age [34]. These data were anonymized, and a specific code was given for every case.

For every included child, we obtained plain X-rays of the left hand and wrist for assessment of bone development and skeletal age calculation. Observers preferred to assess the left side of each subject as done in previous literatures to avoid any observer bias [16, 31, 35]. Moreover, an orthopantomogram was conducted to assess teeth development and calculate dental age. Moreover, Then, all radiographs were scored independently by two experienced and pre-calibrated examiners (M.S.G and S.S.E) who were blinded to the children’s chronological ages.

### Skeletal age assessment using the Fishman method

Fishman eleven-grade system was used to evaluate the maturational patterns of the indicators in the handwrist. Eleven discrete adolescent skeletal maturational indicators (SMI 1- SMI 11) covering the entire period of adolescent development are found on the six anatomical sites located in the thumb, third finger, fifth finger and radius. Skeletal maturity was converted into skeletal age using specific Table [17].

### Dental age assessment using the Nolla method

**Fig. 1:**
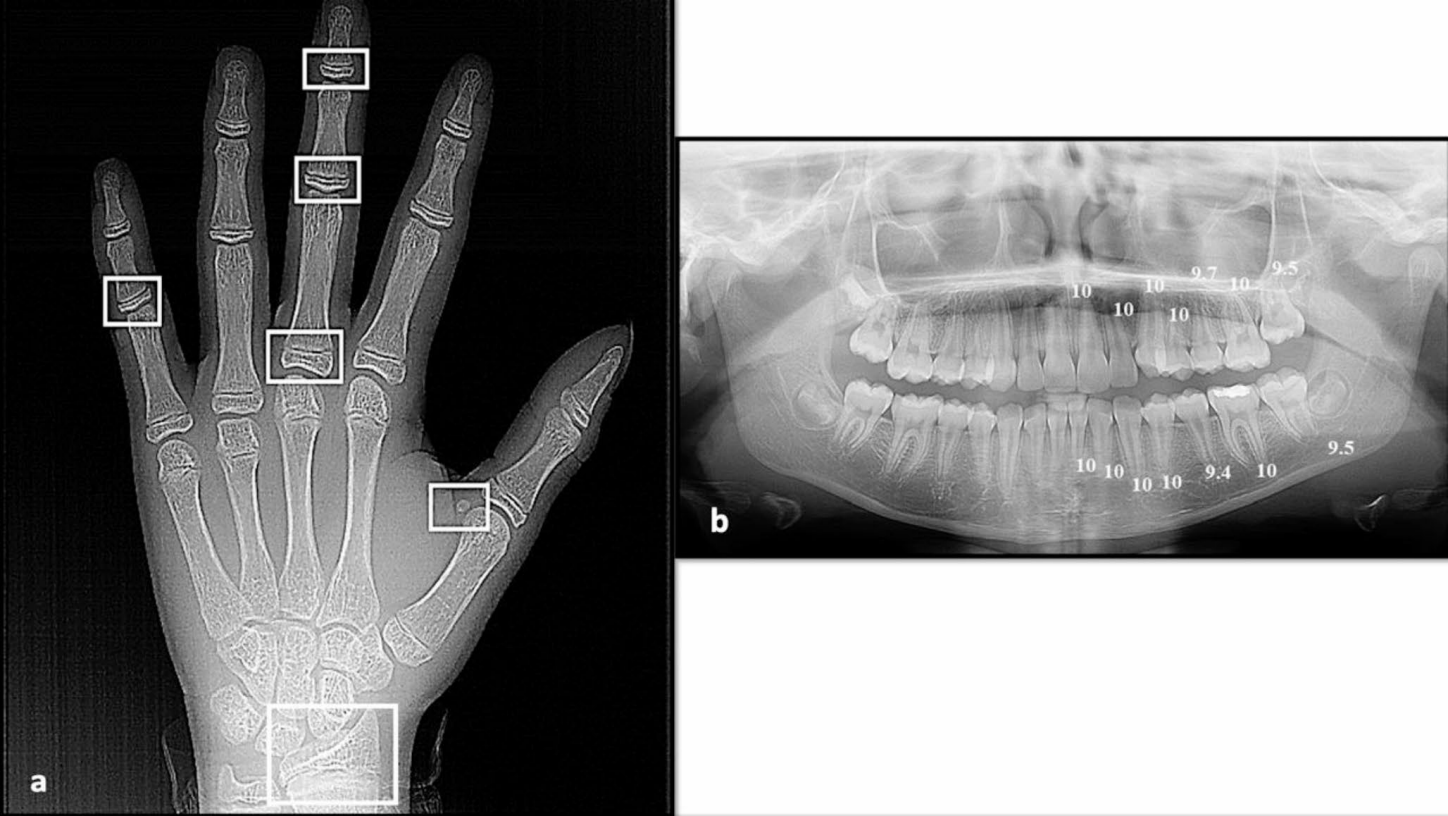
**a**) Hand-wrist X-ray of a 13.22-year female patient showing different sites (in white) of skeletal maturity indicators (SMI) assessed by Fishman method; capping of epiphyses of the middle phalanx on the fifth finger (SMI = 7), and skeletal age is 12.51 years, **b**) Panoramic X-ray of the same patient showed different scoring of dental development of the mandibular and maxillary teeth on the left side (except the third molar), marked according to the Nolla method; the total scoring was 138.1 and the calculated dental age was 13 years

**Fig. 2:**
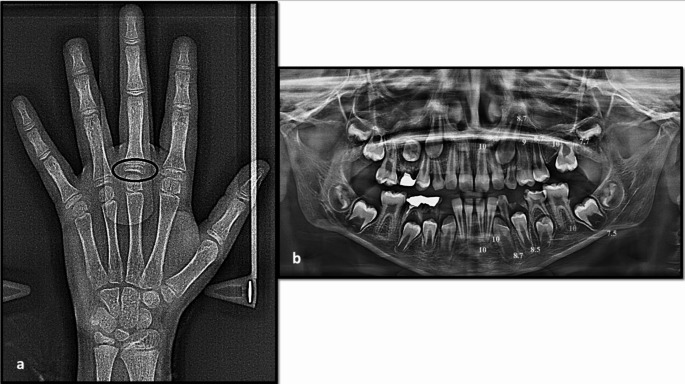
**a**) Hand-wrist X-ray of a 9.05-year female patient showing different sites (in black) of skeletal maturity indicators (SMI) assessed by Fishman method; the proximal phalanx of the third finger showed equal width of the epiphysis and diaphysis (SMI = 1), and skeletal age is 10.23 years, **b**) Panoramic X-ray of the same patient showing different scoring of dental development of the mandibular and maxillary teeth on the left side (except the third molar), marked according to the Nolla method; the total scoring was 118 and the calculated dental age was 9 years

**Fig. 3:**
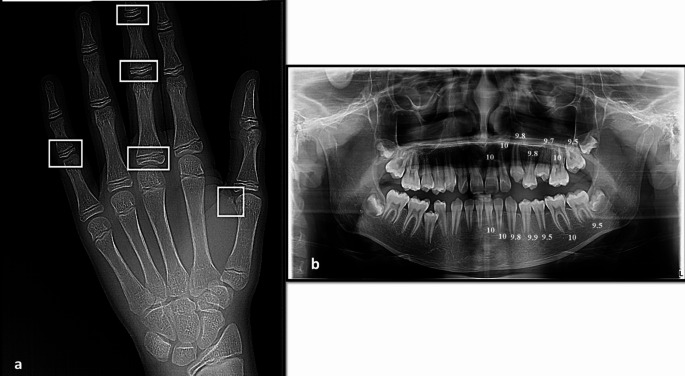
**a**) Hand-wrist X-ray of a 14.98-year male patient showing different sites (in white) of skeletal maturity indicators (SMI) assessed by Fishman method; capping of epiphyses of the middle phalanx on the fifth finger (SMI = 7), and skeletal age is 14.43 years, **b**) Panoramic X-ray of the same patient showing different scoring of dental development of the mandibular and maxillary teeth on the left side (except the third molar), marked according to the Nolla method; the total scoring was 137.5 and the calculated dental age was 14 years

Panoramic images were processed by a computer-aided drafting program (Adobe Photoshop CS, Adobe Systems Incorporated, San Jose, CA, USA) and then matched as closely as possible with the comparative figure of Nolla. The development of the seven left permanent maxillary and mandibular teeth, excluding the third molar, was scored as ‘0’ for the absence of crown, and ‘1’ to ‘10’, depending on the stage of calcification where the stage 10 illustrates that mineralization at the apical end of the root is completed. The sum of stages for 14 maxillary and mandibular teeth for each participant was assigned as Nolla score and graded as (A to H) for males and (a to h) for females using the corresponding tables described by Nolla [36]. Then, the total sum was matched to the average sum for males or females to translate the developmental value into the dental age. Figures 1, 2 and 3 depict the chronological age assessment in three children, showing different skeletal, dental and chronological ages.

### Inter- and intra-observer agreements

Both observers recorded the SMIs and Nolla scores. Furthermore, the first observer re-evaluated each score three weeks after the initial assessment. Images that displayed notable discrepancies between the three recorded measurements during these reliability checks were excluded to maintain data consistency and accuracy. This exclusion process was applied only to individual images (*n* = 3) with significant discrepancies in the scoring. After excluding those 3 cases from the total study cohort, all included radiographs were analyzed to assess inter-observer reliability and reproducibility.

### Data analysis

Analyses were conducted using the R Statistical language (version 4.4.1) [37], model summary (version 2.1.1) [38], Metrics (version 0.1.4) [39], rms (version 6.8.1) [40], Bland Altman (version 0.3.1) [41], gtsummary (version 2.0.0) [42] and psych (version 2.4.6.26) [43].The normality of distribution was tested using the Shapiro-Wilk test and the Q-Q plots. As data did not follow the normal distribution in most groups, continuous numerical variables were summarized using the median, interquartile range (IQR; 25th to 75th percentiles), and the range (minimum to maximum values). The comparisons were made between the chronological and estimated ages using the Friedman Rank Sum Test, and the post hoc pairwise comparisons were carried out using the Wilcoxon-signed rank test with Bonferroni adjustment for multiple comparisons. The mean absolute error (MAE) was calculated for each method. The Bland-Altman plots were created to define the limits of agreement.

Inter and intra-observer variability agreement of the status of the SMIs and Nolla scores were tested using weighted Cohen’s Kappa and the intraclass correlation coefficient (ICC), respectively. The results of the weighted Cohen’s Kappa testing were interpreted using the system developed by McHugh as: κ = 0 -0.20: none agreement, κ = 0.21–0.39: minimal agreement, κ = 0.40–0.59: weak agreement, κ = 0.60–0.79: moderate agreement, κ = 0.80–0.90: strong agreement, κ more than 0.90: almost perfect agreement [44]. The agreement between the estimated ages by both methods and the chronological ages was also tested using the ICC, for which coefficients less than 0.5, between 0.5 and 0.75, between 0.75 and 0.9, and greater than 0.9 indicated poor, moderate, good, and excellent reliability, respectively [45]. Simple linear regression models were created for each method separately and for their combination, using the estimated ages at one time and the scores or indices of the method at another time. The best-performing models were identified based on the highest R^2^ and the least Akaike information criteria (AIC) value. A *P*-value < 0.05 was selected to interpret the results of statistical tests.

## Results

The current study enrolled 176 individuals, males and females, equally distributed with no significant variations in chronological ages (*P* = 0.939). The age of the studied subjects ranged between 8.03 and 15.99 years, with a median of 12 years in both males and females. The sample was evenly distributed across age groups from 8 to 8.9 years to 15–15.99 years. There was no statistically significant difference in the distribution of age groups between males and females (*P* > 0.05). This balance minimized the potential biases and facilitated a straightforward analysis without introducing biases related to age or sex.

Estimating the inter and intra-observer agreement for the SMIs assessed by the Fishman method, showing Weighted Cohen’s Kappa of 0.991 and 0.993, respectively, indicating almost perfect agreement. Similarly, ICCs of the Nolla scores were 0.999, with *P* < 0.001 indicating excellent reliability. Thus, data from the first examiner were used for data analysis (Supplementary Table 1).

Table 1 shows that Fishman and Noll Methods showed a close age estimation (median = 11.96 years [11.37–13.03] and 12.00 years [10.00–13.25]), respectively, compared to chronological age. However, looking into the median difference demonstrates that the Nolla method appeared to slightly under-estimate the age (-0.21 [-0.50 to -0.05]) compared to the chronological age, which was slightly over- estimated using the Fishman method (0.17 [-0.72 to 1.27]). Although we observed a significant difference between the estimated age using both Fishman and Nolla methods and the chronological age (*P* = 0.002 and < 0.001, respectively), the Nolla method was slightly more consistent and accurate across participants. That was supported by the lower MAE the Nolla method had compared to the Fishman method in all participants and in separate male and female cohorts. The age group-specific analysis yielded that the Fishman method tended to over-estimate age in younger age groups, especially for ages 8–9, where the differences (D1) were significantly higher compared to Nolla (a difference of 2.93 and 1.95 for males and females aged 8-8.9). Simultaneously, the Nolla method demonstrated more minor differences from chronological age across age groups, particularly for ages 10–15, suggesting more consistent predictions.

**Table 1:** Comparison between chronological age, Fishman skeletal age and Nolla dental age of the studied participants

Characteristics	Chronological age	Fishman skeletal age	Nolla dental age	D1	D2	D3	P1	P2	P3	MAE1	MAE2	MAE3
	*n* = 176^1^	*n* = 176^1^	*n* = 176^1^	*n* = 176^1^	*n* = 176^1^	*n* = 176^1^						
All participants	12.00 [10.02–13.98]	11.96 [11.37–13.03]	12.00 [10.00–13.25]	0.17 [-0.72 to 1.27]	-0.21 [-0.50 to -0.05]	0.30 [-0.25 to 1.23]	0.002*	< 0.001*	< 0.001*	1.059	0.366	1.030
Female	12.00 [10.08–13.97]	11.71 [10.72–12.51]	11.00 [10.00–13.25]	-0.23 [-0.87 to 0.73]	-0.22 [-0.55 to -0.08]	0.04 [-0.49 to 0.89]	0.593	< 0.001*	0.071	0.897	0.386	0.829
Male	12.01 [10.01–13.98]	12.21 [11.78–13.03]	12.00 [10.00–13.25]	0.68 [-0.42 to 1.82]	-0.17 [-0.37 to -0.03]	0.77 [-0.22 to 1.92]	< 0.001*	< 0.001*	< 0.001*	1.221	0.346	1.232
Female age groups (years)												
8–8.99	8.28 [8.15–8.68]	10.23 [10.23–10.23]	8.00 [8.00–8.50]	1.95 [1.79 to 2.09]	-0.15 [-0.42 to 0.04]	2.23 [1.72 to 2.23]	< 0.001*	0.220	< 0.001*	1.905	0.440	2.137
9–9.99	9.40 [9.15–9.78]	10.23 [10.23–10.72]	9.00 [9.00–9.50]	0.92 [0.73 to 1.18]	-0.08 [-0.40 to 0.01]	1.23 [0.87 to 1.23]	< 0.001*	0.207	< 0.001*	0.984	0.197	1.163
10–10.99	10.33 [10.23–10.58]	10.72 [10.48–10.87]	10.00 [10.00–10.00]	0.22 [0.01 to 0.61]	-0.28 [-0.50 to -0.11]	0.72 [0.23 to 0.87]	0.259	0.032*	0.014*	0.338	0.380	0.642
11–11.99	11.15 [11.10–11.22]	10.87 [10.80–11.04]	11.00 [11.00–11.00]	-0.25 [-0.43 to -0.17]	-0.21 [-0.72 to -0.08]	0.04 [-0.13 to 0.51]	0.037*	0.003*	1.000	0.424	0.359	0.277
12–12.99	12.33 [12.09–12.50]	11.71 [11.71–11.96]	12.00 [12.00–12.00]	-0.70 [-0.97 to -0.30]	-0.50 [-0.81 to -0.15]	-0.29 [-0.29 to -0.04]	0.002*	0.003*	0.126	0.631	0.540	0.195
13–13.99	13.24 [13.18–13.46]	12.51 [11.96–12.51]	13.00 [13.00–13.00]	-0.99 [-1.20 to -0.71]	-0.24 [-0.42 to -0.06]	-0.49 [-1.04 to -0.49]	0.003*	0.002*	0.002*	0.985	0.259	0.726
14–14.99	14.13 [14.08–14.24]	13.27 [12.89–13.27]	14.00 [14.00–14.00]	-0.96 [-1.61 to -0.78]	-0.22 [-0.91 to -0.11]	-0.73 [-1.04 to -0.73]	0.002*	< 0.001*	0.010*	1.113	0.364	0.911
15–15.99	15.21 [15.05–15.76]	14.89 [13.97–14.89]	15.00 [15.00–15.00]	-0.85 [-1.07 to -0.12]	-0.74 [-0.98 to -0.12]	-0.11 [-0.74 to -0.03]	0.036*	< 0.001*	0.165	0.795	0.548	0.582
Male age groups (years)												
8–8.99	8.62 [8.23–8.83]	11.37 [11.37–11.37]	8.00 [8.00–8.00]	2.93 [2.60 to 3.15]	-0.23 [-0.73 to -0.12]	3.37 [3.37 to 3.37]	< 0.001*	0.029*	< 0.001*	2.881	0.470	3.147
9–9.99	9.52 [9.25–9.69]	11.37 [11.37–11.65]	9.00 [9.00–9.00]	1.99 [1.83 to 2.27]	-0.38 [-0.54 to -0.02]	2.37 [2.37 to 2.37]	< 0.001*	0.029*	< 0.001*	2.030	0.426	2.247
10–10.99	10.20 [10.16–10.54]	11.92 [11.65–11.92]	10.00 [10.00–10.50]	1.35 [1.22 to 1.73]	-0.12 [-0.61 to 0.43]	1.92 [0.92 to 1.92]	< 0.001*	0.219	0.002*	1.433	0.591	1.615
11–11.99	11.19 [11.07–11.27]	12.21 [11.92–12.39]	11.00 [11.00–11.00]	1.04 [0.72 to 1.20]	-0.12 [-0.20 to -0.01]	1.21 [0.92 to 1.21]	0.001*	0.029*	0.004*	1.077	0.212	1.091
12–12.99	12.25 [12.10–12.45]	12.57 [12.21–12.75]	12.00 [12.00–12.00]	0.19 [-0.06 to 0.50]	-0.15 [-0.27 to -0.02]	0.21 [0.21 to 0.75]	0.438	0.029*	0.012*	0.355	0.249	0.409
13–13.99	13.21 [13.14–13.24]	12.75 [12.75–13.03]	13.00 [13.00–13.00]	-0.33 [-0.46 to -0.22]	-0.15 [-0.23 to -0.03]	-0.25 [-0.25 to 0.03]	0.009*	0.002*	0.545	0.351	0.161	0.206
14–14.99	14.11 [14.03–14.20]	13.03 [13.03–13.78]	14.00 [14.00–14.00]	-0.98 [-1.02 to -0.40]	-0.11 [-0.21 to -0.02]	-0.25 [-0.97 to -0.22]	0.024*	0.003*	0.101	0.848	0.257	0.669
15–15.99	15.18 [15.08–15.44]	14.43 [14.43–14.43]	15.00 [15.00–15.00]	-0.75 [-0.89 to -0.61]	-0.21 [-0.76 to -0.12]	-0.57 [-0.57 to -0.22]	0.004*	< 0.001*	0.101	0.797	0.397	0.469

n: number; ^1^ Data is presented as median and interquartile range [IQR]; D1: Difference between Fishman skeletal age and chronological age; D2: Difference between Nolla dental age and chronological age; D3: Difference between Nolla dental age and Fishman skeletal age; *P*1: Wilcoxon signed rank test between chronological age & Fishman skeletal age; *P*2: Wilcoxon signed rank test between chronological age & Nolla dental age; *P*3: Wilcoxon signed rank test between Nolla dental age & Fishman skeletal age; MAE1: mean absolute error Fishman; MAE 2: mean absolute error Nolla; MAE3: mean absolute error Nolla vs. Fishman; *: significant at *P* < 0.05

**Fig. 4:**
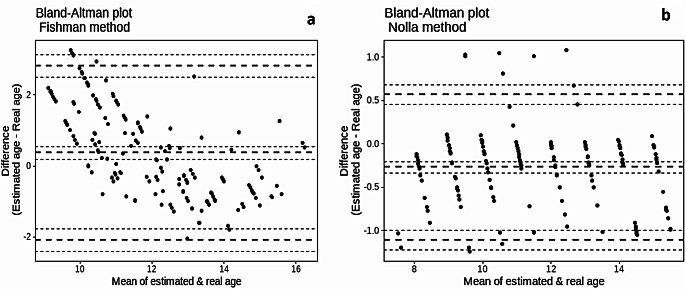
Bland and Altman plots showing differences between **a**) Fishman skeletal age and chronological age in all participants, **b**) Nolla dental age and chronological age in all participants. The middle bold dashed line represents the mean difference, and the two extreme bold dashed lines define the limits of agreement

Figure 4 illustrates that the Fishman method showed greater variability in its estimates, particularly for younger individuals, with a tendency to overestimate age (showing differences greater than + 2 years), while for older ages, it was more accurate or even slightly underestimates. The Nolla method seemed to have better agreement with the actual age (showing differences around 0), as it afforded more consistent and accurate estimates on a broader range of ages, with fewer extreme differences. Table 2 shows that 47.7% of the studied individuals showed SMI between 1 and 3. Regarding males, there were no significant variations between the chronological age and the Fishman skeletal age in boys with SMI above 3. Similarly, females with SMI between 3 and 5 and above 8 showed comparable skeletal chronological ages (*P* > 0.05). In early SMI Stages (1–2), the Fishman method significantly overestimated the chronological age. In contrast, this method significantly underestimates chronological age in females in later SMI Stages (6–8). For males, the Fishman skeletal age often closely approximated the chronological age, with no significant differences in most stages. Moreover, it was observed that females approached the different skeletal maturation stages at earlier ages compared to males of the same SMI.

**Table 2:** Comparison between chronological age and Fishman skeletal age of the studied participants according to SMI in both gender

SMI	Female					Male				
	*n*	Chronological age Median [IQR] (min-max)	Fishman skeletal age Median [IQR] (min-max)	Test Statistic^1^	*P*-value	*n*	Chronological age Median [IQR] (min-max)	Fishman skeletal age Median [IQR] (min-max)	Test Statistic^1^	*P*-value
1	20	9.03 [8.24 to 9.55] (8.03–11.02)	10.23 [10.23 to 10.23] (10.23–10.23)	10.500	< 0.001*	22	9.06 [8.62 to 9.64] (8.12–11.02)	11.37 [11.37 to 11.37] (11.37–11.37)	4.107	< 0.000*
2	10	10.20 [9.21 to 10.50] (8.93–11.15)	10.72 [10.72 to 10.72] (10.72–10.72)	6.000	0.027*	14	10.35 [9.95 to 10.61] (8.99–11.28)	11.92 [11.92 to 11.92] (11.92–11.92)	3.296	< 0.000*
3	8	10.87 [10.08 to 11.17] (9.95–11.72)	10.87 [10.87 to 10.87] (10.87–10.87)	15.000	0.742	10	11.64 [11.12 to 12.05] (10.99–12.36)	12.21 [12.21 to 12.21] (12.21–12.21)	3.000	0.014*
4	5	11.23 [11.21 to 12.01] (10.98–12.02)	11.04 [11.04 to 11.04] (11.04–11.04)	14.000	0.125	13	13.03 [12.25 to 13.21] (11.19–14.01)	12.75 [12.75 to 12.75] (12.57–12.75)	51.000	0.735
5	7	12.15 [11.14 to 12.50] (10.79–12.81)	11.71 [11.71 to 11.71] (11.71–11.71)	19.000	0.446	10	13.69 [13.14 to 14.04] (11.99–14.13)	13.03 [13.03 to 13.03] (13.03–13.03)	42.000	0.160
6	8	13.10 [12.51 to 13.25] (12.26–14.01)	11.96 [11.96 to 11.96] (11.96–11.96)	36.000	0.008*	5	14.18 [14.11 to 14.21] (12.98–15.01)	13.78 [13.78 to 13.78] (13.78–13.78)	11.000	0.438
7	10	13.33 [13.02 to 13.71] (11.98–14.12)	12.51 [12.51 to 12.51] (12.51–12.51)	51.000	0.014*	11	15.12 [14.98 to 15.21] (11.92–15.55)	14.43 [14.43 to 14.43] (14.43–14.43)	54.000	0.067
8	8	14.23 [14.09 to 14.60] (14.02–15.05)	13.27 [13.27 to 13.27] (13.26–13.27)	36.000	0.008*	2	15.88 [15.76 to 15.99] (15.76–15.99)	15.19 [15.19 to 15.19] (15.19–15.19)	3.000	0.500
9	3	15.04 [15.03 to 15.12] (15.03–15.12)	13.97 [13.97 to 13.97] (13.97–13.97)	6.000	0.250	0	-	-	-	-
10	7	15.21 [14.91 to 15.74] (13.95–15.77)	14.89 [14.89 to 14.89] (14.89–14.89)	21.000	0.297	1	14.91 [14.91 to 14.91] (14.91–14.91)	16.17 [16.17 to 16.17] (16.17–16.17)	0.000	1.000
11	2	15.92 [15.86 to 15.98] (15.86–15.98)	16.51 [16.51 to 16.51] (16.51–16.51)	0.000	0.500	0	-	-	-	-

n: number; IQR: interquartile range; Min.- Max.: Minimum-Maximum; SMI: Skeletal maturity indicator; ^1^Wilcoxon signed rank test with continuity correction; *: significant at *P* < 0.05

A comparison of chronological age and Nolla dental age across different Nolla scores for both female and male participants is shown in Table 3. Generally, the Nolla method underestimated the chronological age, with the degree of significance varying across stages and sexes. However, there were no significant variations between the chronological age and Nolla dental age in males, showing Nolla scores between 121 and 135.1 (*P* > 0.05). Nevertheless, based on the findings above, chronological age estimation using the dental and skeletal maturation showed better results closer to the actual age among boys aged between 10 and 13 years (Nolla score) and above SMI 3 than girls.

**Table 3:** Comparison between chronological age and Nolla dental age of the studied participants according to Nolla scores in both gender

Female						Male					
**Nolla Score**	**n**	**Chronological age** **Median [IQR]** **(min-max)**	**Nolla dental age** **Median [IQR]** **(min-max)**	**Test Statistic** ^*1*^	***P*** **-value**	**Nolla Score**	**n**	**Chronological age Median [IQR]** **(min-max)**	**Nolla dental age Median [IQR]** **(min-max)**	**Test Statistic** ^*1*^	***P*** **-value**
**e (101.9)**	2	8.12 [8.03 to 8.20] (8.03–8.20)	7.00 [7.00 to 7.00] (7.00–7.00)	3.000	0.500	**E (94.2)**	0	-	-	-	-
**f (112.3)**	6	8.22 [8.14 to 8.36] (8.12–8.42)	8.00 [8.00 to 8.00] (8.00–8.00)	21.000	0.031*	**F (104.5)**	9	8.26 [8.22 to 8.73] (8.12–8.91)	8.00 [8.00 to 8.00] (8.00–8.00)	45.000	0.004*
**g (118)**	11	9.21 [9.01 to 9.50] (8.93–10.24)	9.00 [9.00 to 9.00] (9.00–9.00)	60.000	0.014*	**G (113.3)**	12	9.51 [9.11 to 9.69] (8.89–10.20)	9.00 [9.00 to 9.00] (9.00–9.00)	74.500	0.006*
**h (127.7)**	14	10.25 [9.99 to 10.50] (8.97–11.15)	10.00 [10.00 to 10.00] (10.00–10.00)	86.000	0.035*	**H (121)**	8	10.10 [9.97 to 10.51] (8.99–10.61)	10.00 [10.00 to 10.00] (10.00–10.00)	25.000	0.383
**i (130.3)**	12	11.18 [11.07 to 11.48] (10.79–12.02)	11.00 [11.00 to 11.00] (11.00–11.00)	70.000	0.017*	**I (126.6)**	12	11.07 [10.79 to 11.20] (9.95–11.28)	11.00 [11.00 to 11.00] (11.00–11.00)	45.000	0.666
**j (135.7)**	9	12.35 [12.15 to 12.50] (11.98–12.81)	12.00 [12.00 to 12.00] (12.00–12.00)	43.000	0.018*	**J (131.6)**	11	12.15 [12.02 to 12.27] (10.99–12.95)	12.00 [12.00 to 12.00] (12.00–12.00)	54.000	0.068
**k (138.1)**	11	13.24 [13.06 to 13.42] (12.33–13.71)	13.00 [13.00 to 13.00] (13.00–13.00)	56.000	0.045*	**K (135.1)**	14	13.15 [13.01 to 13.23] (11.92–14.01)	13.00 [13.00 to 13.00] (13.00–13.00)	77.000	0.132
**l (139.1)**	14	14.18 [14.05 to 14.91] (13.95–15.05)	14.00 [14.00 to 14.00] (13.00–14.00)	102.500	0.002*	**L (137.5)**	11	14.11 [14.02 to 14.21] (13.97–15.01)	14.00 [14.00 to 14.00] (14.00–14.00)	63.000	0.005*
**m (139.6)**	9	15.22 [15.12 to 15.77] (15.01–15.98)	15.00 [15.00 to 15.00] (15.00–15.00)	45.000	0.004*	**M (139)**	11	15.18 [15.04 to 15.55] (14.91–15.99)	15.00 [15.00 to 15.00] (15.00–15.00)	63.000	0.005*

n: number; IQR: interquartile range; Min.- Max.: Minimum-Maximum; e-m: Nolla scores for female; E-M: Nolla scores for male; ^1^Wilcoxon signed rank test with continuity correction; *: significant at *P* < 0.05

Table 4 and 5 depict the association between Fishman SMI and Nolla Dental Score in females and males. There was a strong association between dental development (Nolla scores) and skeletal maturation (SMI), with both measures showing parallel progress toward full maturation in both females and males. The females showed a clear progression from early Nolla scores (e, f, g), associated with early SMI stages (SMI 1), to later Nolla scores (l) associated with higher SMIs (SMI 8, SMI 9, SMI 10, SMI 11). In males, a similar trend was observed with early Nolla scores (F, G) primarily associated with SMI 1, and later Nolla stage (M) corresponding to higher SMI stages (SMI 7, SMI 8, SMI 10).

**Table 4:** The association between Fishman skeletal maturity indicators and Nolla dental score (e-m) in females

Nolla Score	SMI in female participants										
	SMI 1	SMI 2	SMI 3	SMI 4	SMI 5	SMI 6	SMI 7	SMI 8	SMI 9	SMI 10	SMI 11
e (101.9)	2 (10%)	0 (0%)	0 (0%)	0 (0%)	0 (0%)	0 (0%)	0 (0%)	0 (0%)	0 (0%)	0 (0%)	0 (0%)
f (112.3)	6 (30%)	0 (0%)	0 (0%)	0 (0%)	0 (0%)	0 (0%)	0 (0%)	0 (0%)	0 (0%)	0 (0%)	0 (0%)
g (118)	9 (45%)	0 (0%)	0 (0%)	2 (20%)	0 (0%)	0 (0%)	0 (0%)	0 (0%)	0 (0%)	0 (0%)	0 (0%)
h (127.7)	3 (15%)	0 (0%)	0 (0%)	7 (70%)	4 (50%)	0 (0%)	0 (0%)	0 (0%)	0 (0%)	0 (0%)	0 (0%)
i (130.3)	0 (0%)	0 (0%)	0 (0%)	1 (10%)	4 (50%)	5 (100%)	2 (29%)	0 (0%)	0 (0%)	0 (0%)	0 (0%)
j (135.7)	0 (0%)	0 (0%)	0 (0%)	0 (0%)	0 (0%)	0 (0%)	5 (71%)	3 (38%)	1 (10%)	0 (0%)	0 (0%)
k (138.1)	0 (0%)	0 (0%)	0 (0%)	0 (0%)	0 (0%)	0 (0%)	0 (0%)	4 (50%)	7 (70%)	0 (0%)	0 (0%)
l (139.1)	0 (0%)	2 (29%)	0 (0%)	0 (0%)	0 (0%)	0 (0%)	0 (0%)	1 (13%)	2 (20%)	8 (100%)	1 (33%)
m (139.6)	0 (0%)	5 (71%)	2 (100%)	0 (0%)	0 (0%)	0 (0%)	0 (0%)	0 (0%)	0 (0%)	0 (0%)	2 (67%)

SMI: Skeletal maturity indicator; e-m: Nolla scores for female

**Table 5:** The association between Fishman skeletal maturity indicators and Nolla dental score (F-M) in males

Nolla score	SMI in male participants								
	SMI 1	SMI 2	SMI 3	SMI 4	SMI 5	SMI 6	SMI 7	SMI 8	SMI 9
F (104.5)	9 (41%)	0 (0%)	0 (0%)	0 (0%)	0 (0%)	0 (0%)	0 (0%)	0 (0%)	0 (0%)
G (113.3)	11 (50%)	1 (7.1%)	0 (0%)	0 (0%)	0 (0%)	0 (0%)	0 (0%)	0 (0%)	0 (0%)
H (121)	1 (4.5%)	7 (50%)	0 (0%)	0 (0%)	0 (0%)	0 (0%)	0 (0%)	0 (0%)	0 (0%)
I (126.6)	1 (4.5%)	6 (43%)	4 (40%)	1 (7.7%)	0 (0%)	0 (0%)	0 (0%)	0 (0%)	0 (0%)
J (131.6)	0 (0%)	0 (0%)	6 (60%)	4 (31%)	1 (10%)	0 (0%)	0 (0%)	0 (0%)	0 (0%)
K (135.1)	0 (0%)	0 (0%)	0 (0%)	8 (62%)	4 (40%)	1 (20%)	1 (9.1%)	0 (0%)	0 (0%)
L (137.5)	0 (0%)	0 (0%)	0 (0%)	0 (0%)	5 (50%)	4 (80%)	2 (18%)	0 (0%)	0 (0%)
M (139)	0 (0%)	0 (0%)	0 (0%)	0 (0%)	0 (0%)	0 (0%)	8 (73%)	2 (100%)	1 (100%)

SMI: Skeletal maturity indicator; F-M: Nolla scores for male

**Fig. 5:**
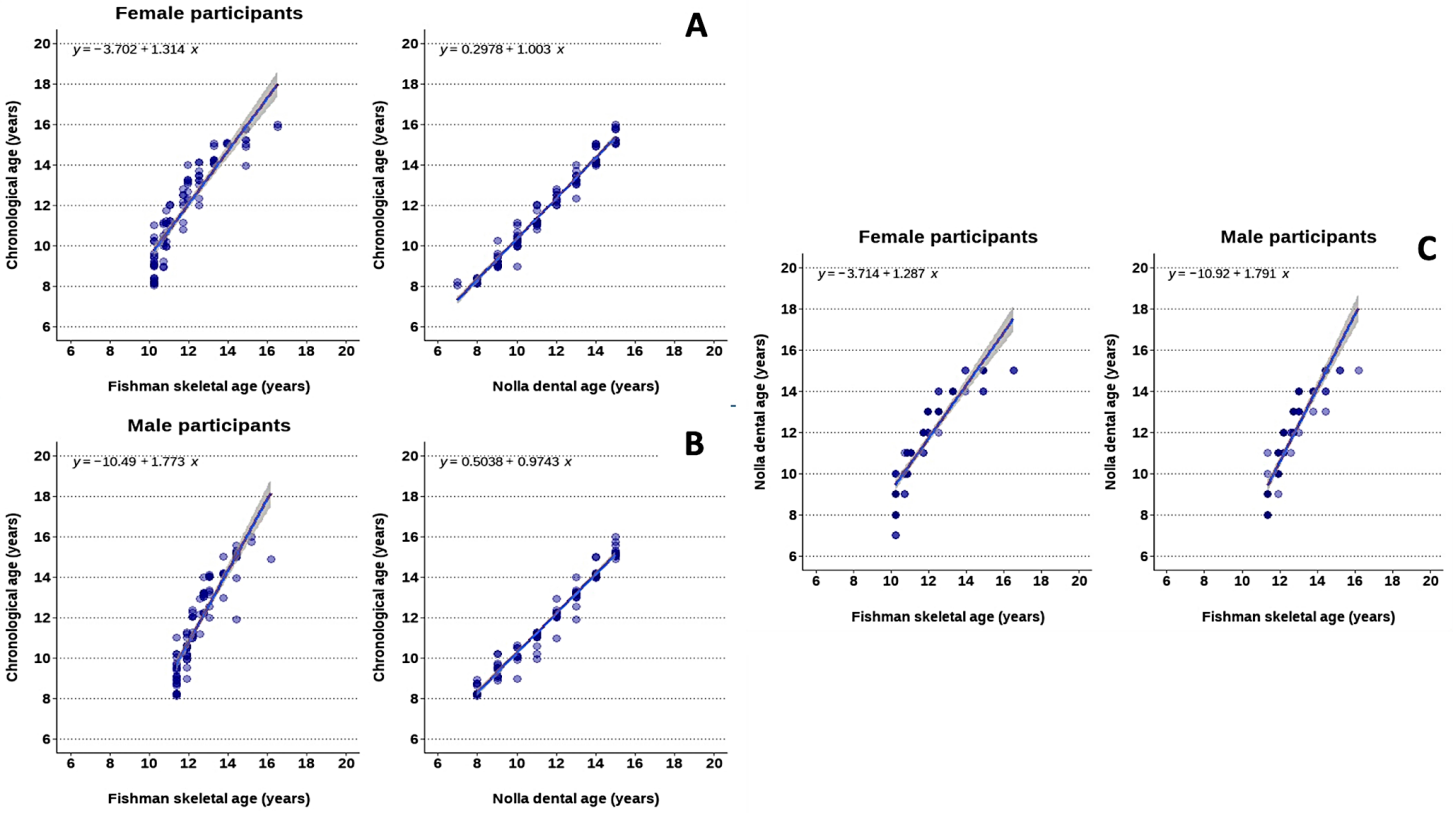
Graphical representation of the Spearman correlation between **a**) The chronological age and both of Fishman skeletal age and Nolla dental age in female participants, **b**) The chronological age and both of Fishman skeletal age and Nolla dental age in male participants, **c**) The Fishman skeletal age and Nolla dental age in both female and male participants

Inspecting Fig. 5 conveys that, among females and males, there was a significant positive correlation between chronological age, Fishman skeletal age, and Nolla dental age. A similar correlation was observed between Fishman skeletal age and Nolla dental age (*P* < 0.001). Correlating the estimated age using the Fishman method and the chronological age showed an ICC of 0.854 (0.786–0.902) and 0.660 (0.524–0.763) for females and males, respectively. Similarly, the ICC between the estimated age using the Nolla method and the chronological age were 0.973 (0.96–0.982) and 0.977 (0.965–0.985) for females and males, respectively (*P* < 0.001). The estimated age by the Fishman method was positively correlated with the corresponding age assessed by the Nolla method among females (ICC of 0.846, 95% CI = 0.774–0.896) and males (ICC of 0.618, 95% CI = 0.471, 0.732). The ICC analyses elucidated that the Nolla method exhibited better ICC coefficients than the Fishman method.

Table 6 displays four models established for predicting the chronological age in females. The model adopting the Fishman skeletal age significantly estimated chronological age. For each unit increase in skeletal age, chronological age increased by 1.314 years. This model explained about 82.9% variance in chronological age. The other model using the Nolla dental age was another significant predictor of chronological age, with each unit increase in dental age corresponding to a nearly identical increase in chronological age (1.003 years), and R^2^ = 0.969. The low AIC = 96 suggests a better fit than the skeletal age model. The third model adopted specific weights for different stages of skeletal maturity (SMI stages 2 to 11), and it explained about 91.7% of the variance. Its performance is midway better than the first model (skeletal age) but worse than the second model (dental age). The fourth model using different Nolla scores, “Chronological age = 8.115 + weighted values for Nolla scores (f to m)”, showed the best performance among the four established models, the highest R² (0.973) and low AIC (96), indicating this model fitted the data best.

**Table 6:** Linear regression analysis for the prediction of the chronological age in female participants from both studied methods

Models of female participants	Predictors	B	SE	95% CI	t	*P*-value	*R* ^2^	AIC	Equation to predict chronological age
Model (skeletal age)	Intercept	-3.702	0.769	-5.230, -2.174	-4.816	< 0.001*	0.829	245	-3.702 + 1.314 x Fishman skeletal age
	Fishman skeletal age	1.314	0.064	1.186, 1.442	20.421	< 0.001*			
Model (dental age)	Intercept	0.298	0.229	-0.157, 0.753	1.301	0.197	0.969	96	0.298 + 1.003 x Nolla dental age
	Nolla dental age	1.003	0.020	0.965, 1.042	51.448	< 0.001*			
Model (SMI)	Intercept	9.071	0.157	8.758, 9.383	57.856	< 0.001*	0.917	199	9.071+ 0.982 x SMI 2 + 1.667 X SMI 3 + 2.420 x SMI 4 + 2.915 x SMI 5 + 3.926 x SMI 6 + 4.194 x SMI 7 + 5.292 x SMI 8 + 5.993 x SMI 9 + 6.045 x SMI 10 + 6.850 x SMI 11
	SMI 2	0.982	0.272	0.441, 1.522	3.614	0.001*			
	SMI 3	1.667	0.293	1.083, 2.251	5.684	< 0.001*			
	SMI 4	2.420	0.351	1.721, 3.118	6.902	< 0.001*			
	SMI 5	2.915	0.308	2.302, 3.528	9.468	< 0.001*			
	SMI 6	3.926	0.293	3.342, 4.510	13.385	< 0.001*			
	SMI 7	4.194	0.272	3.654, 4.735	15.447	< 0.001*			
	SMI 8	5.292	0.293	4.708, 5.876	18.043	< 0.001*			
	SMI 9	5.993	0.434	5.128, 6.857	13.805	< 0.001*			
	SMI 10	6.045	0.308	5.432, 6.658	19.633	< 0.001*			
	SMI 11	6.850	0.520	5.814, 7.885	13.173	< 0.001*			
Model (Nolla score)	Intercept	8.115	0.279	7.560, 8.670	29.108	< 0.001*	0.973	96	8.115 + 0.130 x f + 1.176 x g + 2.153 x h + 3.182 x i + 4.243 x j + 5.079 x k + 6.244 x l + 7.324 x m
	f	0.130	0.322	-0.511, 0.771	0.404	0.687			
	g	1.176	0.303	0.573, 1.779	3.880	< 0.001*			
	h	2.153	0.298	1.560, 2.746	7.223	< 0.001*			
	i	3.182	0.301	2.582, 3.781	10.566	< 0.001*			
	j	4.243	0.308	3.629, 4.856	13.766	< 0.001*			
	k	5.079	0.303	4.475, 5.682	16.757	< 0.001*			
	l	6.244	0.298	5.651, 6.838	20.951	< 0.001*			
	m	7.324	0.308	6.710, 7.937	23.762	< 0.001*			

SMI: Skeletal maturity indicator; f-m: Nolla scores for female; B: regression coefficient; SE: standard error of B; CI: confidence interval; AIC: Akaike information criteria; *: significant at *P* < 0.05

Table 7 shows four models that adopted different predictors: The Fishman skeletal age, Nolla dental age, Skeletal Maturity Index (SMI), and Nolla score. The model incorporating only the Nolla score was the most accurate predictor of chronological age in male participants, owing to its highest R² (0.968) and lowest AIC (106). The other dental age model also performed well, with a slightly lower R² (0.962). Nevertheless, the model incorporating the SMI performed well (R² = 0.899), while the model using the skeletal age showed the least predictive accuracy (R² = 0.816) compared to the other models.

**Table 7:** Linear regression analysis for the prediction of the chronological age in male participants from both studied methods

Models of male participants	Predictors	B	SE	95% CI	t	*P*-value	*R* ^2^	AIC	Equation to predict chronological age
Model (skeletal age)	Intercept	-10.487	1.148	-12.769, -8.205	-9.135	< 0.001*	0.816	248	-10.487 + 1.773 x Fishman skeletal age
	Fishman skeletal age	1.773	0.091	1.593, 1.953	19.538	< 0.001*			
Model (dental age)	Intercept	0.504	0.249	0.009, 0.999	2.022	0.046*	0.962	110	0.504 + 0.974 x Nolla dental age
	Nolla dental age	0.974	0.021	0.933, 1.016	46.394	< 0.001*			
Model (SMI)	Intercept	9.155	0.160	8.837, 9.473	57.363	< 0.001*	0.899	209	9.155 + 1.162 x SMI 2 + 2.469 x SMI 3 + 3.670 x SMI 4 + 4.298 x SMI 5 + 4.943 x SMI 6 + 5.616 x SMI 7 + 6.720 x SMI 8 + 5.755 x SMI 10
	SMI 2	1.162	0.256	0.653, 1.672	4.541	< 0.001*			
	SMI 3	2.469	0.285	1.901, 3.037	8.648	< 0.001*			
	SMI 4	3.670	0.262	3.149, 4.192	14.016	< 0.001*			
	SMI 5	4.298	0.285	3.730, 4.866	15.054	< 0.001*			
	SMI 6	4.943	0.371	4.205, 5.681	13.328	< 0.001*			
	SMI 7	5.616	0.276	5.066, 6.166	20.316	< 0.001*			
	SMI 8	6.720	0.553	5.620, 7.820	12.155	< 0.001*			
	SMI 10	5.755	0.765	4.232, 7.278	7.519	< 0.001*			
Model (Nolla score)	Intercept	8.450	0.140	8.172, 8.728	60.518	< 0.001*	0.968	106	8.450 + 1.036 x G + 1.642 x H + 2.463 x I + 3.663 x J + 4.635 x K + 5.796 x L + 6.847 x M
	G	1.036	0.185	0.668, 1.403	5.608	< 0.001*			
	H	1.642	0.204	1.237, 2.048	8.070	< 0.001*			
	I	2.463	0.185	2.095, 2.830	13.332	< 0.001*			
	J	3.663	0.188	3.288, 4.037	19.454	< 0.001*			
	K	4.635	0.179	4.279, 4.991	25.899	< 0.001*			
	L	5.796	0.188	5.422, 6.171	30.787	< 0.001*			
	M	6.847	0.188	6.473, 7.222	36.369	< 0.001*			

SMI: Skeletal maturity indicator; G-M: Nolla scores for male; B: regression coefficient; SE: standard error of B; CI: confidence interval; AIC: Akaike information criteria; *: significant at *P* < 0.05

Table 8 shows that among both sexes, combining the Nolla score and SMI in one model did not yield superior performance compared to using the Nolla score alone. The model incorporating skeletal maturation indicators, and dental developmental scores exhibited R² of 0.971, an AIC of 101 in females and R² of 0.968 and an AIC of 106 in males. These values were slightly lower or equal to the performances adopted by the model using the Nolla score only. Besides, the SMI contribution in the combined model was minimal and did not significantly enhance prediction accuracy over dental mineralization alone. This suggests that, in this dataset, the Nolla score was a more robust and precise age predictor.

**Table 8:** Linear regression analysis for the model predicting the chronological age using SMI and Nolla score in both sexes

Parameters included in the model (SMI and Nolla score)	Predictors	B	SE	95% CI	t	*P*-value	*R* ^2^	AIC	Equation to predict chronological age
Female	Intercept	8.115	0.274	7.568, 8.662	29.618	< 0.001*	0.971	101	8.115–0.333 x SMI 2–0.286 x SMI 3–0.041 x SMI 4 -0.566 x SMI 5–0.637 x SMI 6–0.682 x SMI 7–0.384 x SMI 8–0.130 x SMI 9–0.110 x SMI 10 + 0.503 x SMI 11 + 0.130 x f + 1.236 x g + 2.401 x h + 3.416 x i + 4.845 x j + 5.744 x k + 6.632 x l + 7.302 x m
	SMI 2	-0.333	0.199	-0.729, 0.064	-1.671	0.099			
	SMI 3	-0.286	0.253	-0.792, 0.220	-1.129	0.263			
	SMI 4	-0.041	0.335	-0.710, 0.628	-0.124	0.902			
	SMI 5	-0.566	0.397	-1.358, 0.225	-1.427	0.158			
	SMI 6	-0.637	0.477	-1.588, 0.315	-1.335	0.186			
	SMI 7	-0.682	0.496	-1.672, 0.309	-1.373	0.174			
	SMI 8	-0.384	0.548	-1.478, 0.709	-0.701	0.486			
	SMI 9	-0.130	0.603	-1.333, 1.073	-0.216	0.830			
	SMI 10	-0.110	0.583	-1.273, 1.053	-0.188	0.851			
	SMI 11	0.503	0.655	-0.803, 1.809	0.768	0.445			
	f	0.130	0.316	-0.501, 0.761	0.411	0.682			
	g	1.236	0.300	0.638, 1.835	4.121	< 0.001*			
	h	2.401	0.331	1.740, 3.061	7.252	< 0.001*			
	i	3.416	0.397	2.625, 4.208	8.609	< 0.001*			
	j	4.845	0.512	3.823, 5.868	9.456	< 0.001*			
	k	5.744	0.565	4.616, 6.871	10.163	< 0.001*			
	l	6.632	0.597	5.440, 7.823	11.103	< 0.001*			
	m	7.302	0.655	5.996, 8.608	11.156	< 0.001*			
Male	Intercept	8.450	0.134	8.183, 8.717	63.111	< 0.001*	0.968	106	8.450 − 0.067 x SMI 2 + 0.271 x SMI 3 + 0.616 x SMI 4 + 0.240 x SMI 5 + 0.389 x SMI 6 + 0.094 x SMI 7 + 0.768 x SMI 8–0.197 x SMI 10 + 1.041 x G + 1.701 x H + 2.354 x I + 3.269 x J + 4.180x K + 5.529 x L + 6.657 x M
	SMI 2	-0.067	0.247	-0.559, 0.425	-0.270	0.788			
	SMI 3	0.271	0.319	-0.366, 0.907	0.848	0.399			
	SMI 4	0.616	0.361	-0.103, 1.335	1.709	0.092			
	SMI 5	0.240	0.401	-0.560, 1.039	0.598	0.552			
	SMI 6	0.389	0.443	-0.494, 1.272	0.877	0.383			
	SMI 7	0.094	0.459	-0.821, 1.009	0.205	0.838			
	SMI 8	0.768	0.558	-0.345, 1.881	1.375	0.173			
	SMI 10	-0.197	0.626	-1.446, 1.051	-0.315	0.754			
	G	1.041	0.178	0.686, 1.397	5.840	< 0.001*			
	H	1.701	0.291	1.120, 2.281	5.843	< 0.001*			
	I	2.354	0.293	1.770, 2.938	8.040	< 0.001*			
	J	3.269	0.368	2.535, 4.004	8.874	< 0.001*			
	K	4.180	0.403	3.377, 4.984	10.371	< 0.001*			
	L	5.529	0.442	4.649, 6.410	12.518	< 0.001*			
	M	6.657	0.499	5.663, 7.652	13.342	< 0.001*			

SMI: Skeletal maturity indicator; f-m: Nolla scores for female; G-M: Nolla scores for male; B: regression coefficient; SE: standard error of B; CI: confidence interval; AIC: Akaike information criteria; *: significant at *P* < 0.05

**Fig. 6:**
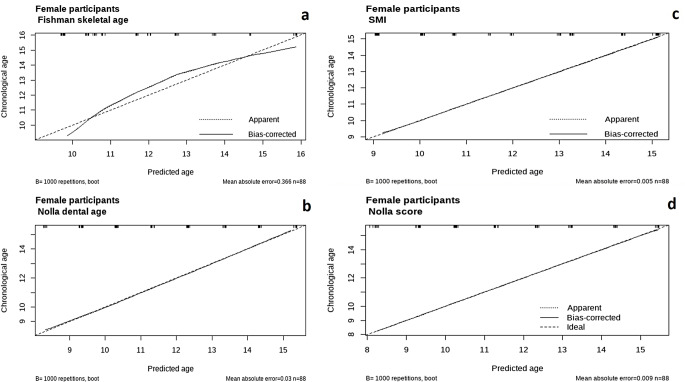
Calibration of the proposed models in female participants **a**) Fishman skeletal age model, **b**) Nolla dental age model, **c**) Skeletal maturity indicator model, **d**) Nolla score model

**Fig. 7:**
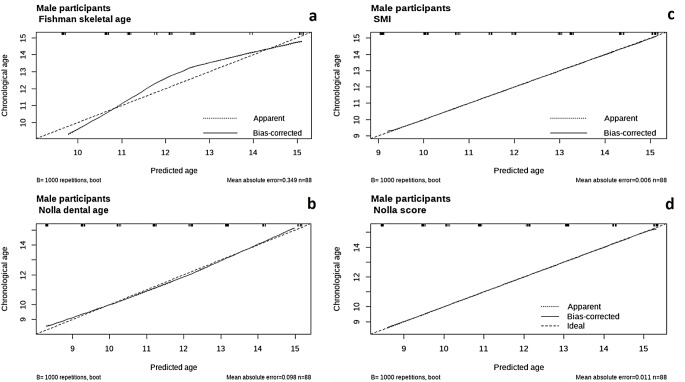
Calibration of the proposed models in male participants **a**) Fishman skeletal age model, **b**) Nolla dental age model, **c**) Skeletal maturity indicator model, **d**) Nolla score model

**Fig. 8:**
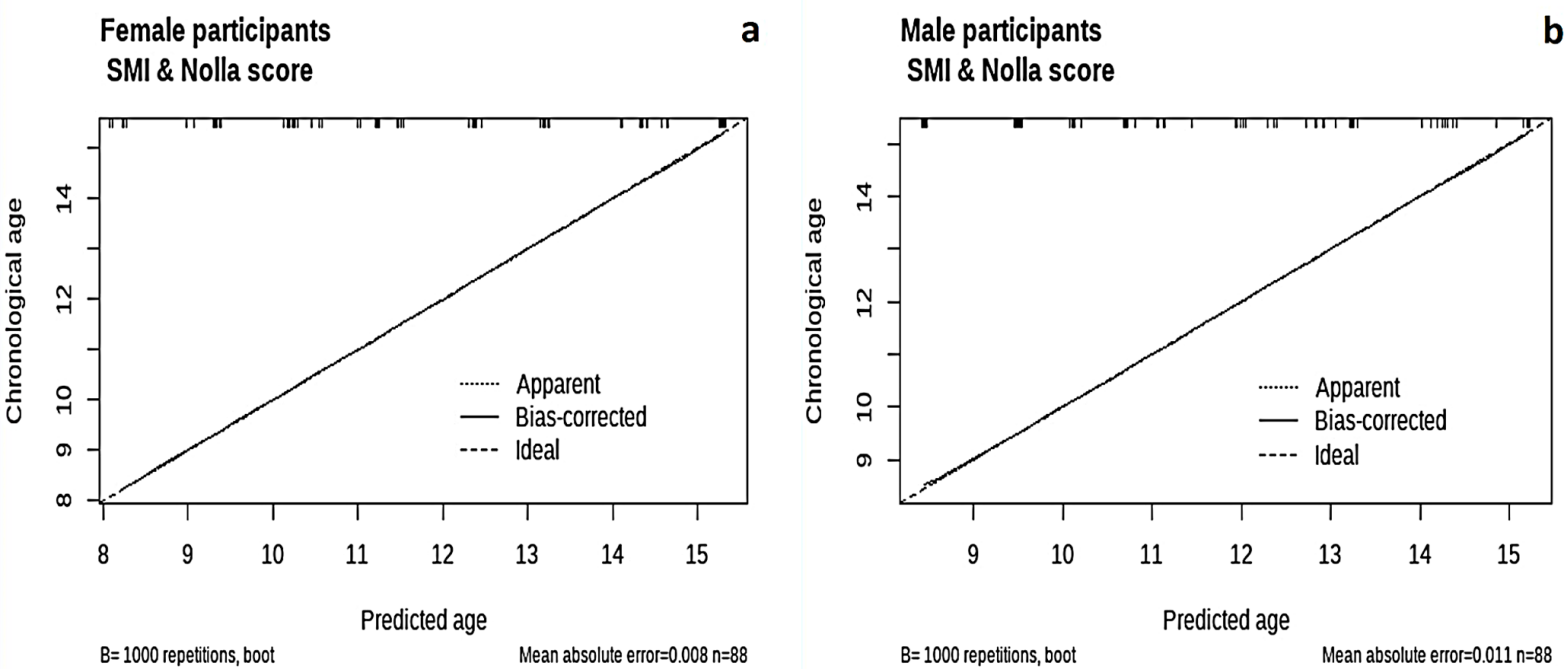
Calibration of the proposed models predicting the chronological age using SMI and Nolla Score in **a**) Female participants, **b**) Male participants

The calibration of the eight proposed models shown in Figs. 6 and 7 shows that the models adopting the Nolla dental age and Nolla score exhibited exceptionally high performances with minimal prediction errors among female and male participants. The model using the SMI showed very close alignment to the ideal prediction line and low mean absolute errors, indicating strong prediction capability. Eventually, the Fishman skeletal age model showed moderate errors (MAE = 0.366 in females versus 0.349 in males), with a tendency to overestimate at younger ages and underestimate at older ages for both males and females. Overall, the models adopting the Nolla score were the most reliable for estimating the chronological age in both male and female participants, followed closely by the models including the Nolla dental age and SMI model. The calibration of the model incorporating SMIs and Nolla scores is shown in Fig. 8, displaying MAE that is approximately identical to the model using the Nolla Score alone (0.008 in females and 0.011 in males).

## Discussion

Despite of its significance, chronological age per se is of low importance and does not reflect the actual developmental stage of children [46]. Thus, more reliable age estimation depends on biological evidence, including somatic, skeletal, dental, and sexual development. There is an increasing demand to establish more reliable age estimation methods with less liability for errors. Combining different methods increases the reliability and decreases the margin of errors, allowing more precise age estimation [26]. No agreement exists on the most accurate and reliable method to estimate chronological age [47]. Although some studies have been aimed at validating the skeletal and dental mineralization for age prediction among different populations, these results showed inconsistent findings [16, 32].

The current study aimed to compare the performance of two established methods showing skeletal maturation and dental mineralization as age predictors. Moreover, to assess if combining both methods would offer more reliable and accurate age estimation. According to our study results, although there were significant differences between the chronological age from one side and the skeletal and dental ages from the other, both adopted methods are still valid age predictors with variable sex and age group accuracy. However, depending on the dental mineralization, the age estimation using the Nolla method showed superior performance compared to skeletal maturation using the Fishman method. However, both methods significantly correlated with the chronological age and each other. Among the proposed models, the obtained findings conveyed that the Nolla score model showed the highest performance, and the lowest errors compared to the other models.

In agreement with the findings obtained, where both investigated methods showed variable errors differed according to age group and sex, previous literature acknowledged that there are generally slight variations between skeletal and dental ages and chronological ages [33]. Ideally, the estimation error correlation coefficient for a perfect age-assessing tool should be 0, denoting a precise estimation for all ages under assessment [48]. However, these ideal situations are seldom met. Pereira et al. assessed five different dental scoring systems (Mincer with 5 stages, Kullman with 7 stages, Demirjian with 8 stages, Köhler with 10 stages, Haaviko with 11 stages, Moorrees, Fanning and Hunt (MFH) with 14 stages, and Gleiser and Hunt with 15 stages) and found that all investigated methods showed statistically significant differences between the estimated age and the chronological age, which support the presence of estimation errors [49]. Nevertheless, great variations of dental and skeletal age from the actual chronological age suggest pathological disorders [33]. Typically, the skeletal age varies from the chronological age by about ± 10% [50]. From the forensic point of view, a difference of one year between the chronological age and dental age was considered acceptable for age estimation by legal authorities [51].

The Fishman method utilizes the SMIs to estimate the chronological age using hand and wrist radiography [17]. The Fishman method showed high accuracy in estimating the age with no significant variations between skeletal age and actual age among Indian children [32]. On the contrary, this method was less accurate in Indonesian and Lebanese children [16, 52]. In line with the present study, the Fishman method overestimated the age, particularly in young children aged around 10 years [30]. Saade et al. criticized the Fishman method for overestimating the age in higher age groups (8–16 years) in the Lebanese population [16], as well as Bhadana et al. in Indian boys and girls [46]. Conversely, although the Fishman method was successfully applied to estimate the chronological age among South Indian children, it displayed an age underestimation with a low mean difference of about 0.4 years in males and 0.3 years in females [32]. Similar findings were obtained elsewhere [30, 31]. Apart from ethnicity, the observed discrepancy in different populations might be attributed to variations in the sample size, socioeconomic standards, and health conditions [30].

The Nolla method, a dental age estimation tool based on radiography of the observed calcification status in permanent teeth, is particularly practical for forensic age estimation [36]. Its high reliability, indicated by the low intra-observer error, and performance comparable to more complex methods, make it a preferred choice [53]. Caro and Contreras reported higher accuracy in age estimation using the Nolla method compared to other methods they examined [54]. Moreover, Nolla method offers two more degrees of mineralization of the crown than the well-known Demirjian method [22, 23].

Due to the very low variability shown by the Nolla method in the present study, as indicated by Bland and Altman plots, this method seemed to be a better predictor for actual age than the Fishman method. This conclusion was rooted in the fact that the preference for the age-identifying method is based on its accuracy and precision. Accuracy stands for *‘‘the closeness of a computed value to its true value’*’ which is indicated in the present study by the difference in the means [55]. At the same time, precision describes the observed variations between the standard and sample population [56]. To our knowledge, no previous studies estimated age using the Nolla method among Egyptian populations. Thus, we can only describe the Nolla method as an accurate rather than precise age-estimating method.

However, the precision of the Nolla method was criticized. Nolla method permits the investigator to opt between 10 different stages but besides offers three inter stage options for each stage, which increase the number of stages to 40, which was shown to decrease precision [57, 58]. Nolla method possesses several other limitations. Involving the arbitrary full root length measurements in one of them. Besides, this method estimates the age in years only without considering months. The Nolla method seems more accurate in older age due to the observed narrower scores with age advancement [31].

Few studies have investigated the Nolla method’s utility in predicting the chronological age amongst different populations [23]. The Nolla method showed an underestimation of age among Turkish, Brazilian, Bangladeshi, British, Caucasian, Portuguese, Spanish, Malaysian, and Yemeni children [11, 22, 23, 31, 56, 59, 60]. Maber et al. reported a mean difference of 0.87 years for boys and 1.18 years for girls, which was higher than the means reported in the current study. They observed that this underestimation was evident in all ages and increased with the advancement of age [56]. The reason behind this underestimation can be attributed to the difficulty in evaluating the small apex opening, which is commonly closed or even fully closed in older ages [48]. Miloglu reported a mean difference of -0.3 years in Turkish children, which is also higher than the total difference in the mean reported in the present study [22]. Inversely, Holtgrave reported age overestimation among European boys [61]. Partially inconsistent with these speculations, Freitas et al. reported age overestimation in young children and age underestimation in older children among Brazilian children [62]. However, the Nolla method accurately estimated the chronological age among European girls [61].

The current study revealed that dental age was more accurate as a chronological age predictor than skeletal age. These results were concordant with those of Mollabashi et al., who owed the superior performance of dental methods to the significant influence of health conditions and environmental alterations on skeletal maturation [63]. Nonetheless, these findings contradicted Cummaudo et al., who mentioned that although the age estimation using radiological methods exhibited large error ranges, these methods remained more reliable and quantifiable than other methods of age estimation [64]. Likewise, Alqadi and Abuaffa found that despite the higher R^2^ for the Demirjian method than the Fishman method for both sexes, the Fishman skeletal age estimation was more accurate than dental in Yemeni female children [30]. The same findings were reported elsewhere [16].

Although we tried to obtain a more precise and accurate age predictor model by combining SMI and Nolla scores, our findings failed to prove the superior performance of this inclusive model compared to the model using Nolla scores alone. Concordant with the present study, Sachan et al. mentioned that there is no need to obtain more radiographs on different bone areas if the patient underwent panoramic radiographs showing the different calcification stages of canines [33]. Apart from being non-invasive, another advantage of using dental mineralization in age estimation is the minimal risk of tissue damage and the low dose of radiation the individual is exposed to radiation [65]. This came in contrast to previous studies that recommended using another tool to assess the developmental maturation of another structure, like the teeth, to confirm the chronological age when skeletal age is consensus reached [16]. The previous literature supported the idea that combining dental and skeletal maturity stages is a reliable tool for age estimation in pediatrics [63, 66, 67].

As per the current study, the skeletal age, assessed by the Fishman method, and the dental age, assessed by several methods, exhibited a significant correlation with the chronological age [46]. Consistently, Alqadi and Abuaffan conveyed similar significant correlations between chronological age and skeletal and dental ages using the same methods adopted in the present study [31]. Flores et al. reported that maxillofacial growth velocity is directly proportional to the SMI in wrist radiography [18]. Unlike some dental age assessing methods, Bhadana et al. denied a significant correlation between the assessed skeletal age using the Fishman method and the chronological age [46]. Nonetheless, chronological age was correlated neither with dental development nor skeletal maturation in healthy Indian children [33].

In line with the present study, literature reported a significant correlation between skeletal age (SMI) assessed by the Fishman method and dental development assessed by different methods, including the Nolla, Demirjian, and Willem’s methods [25, 46, 68]. Sachan et al. reported a correlation coefficient of 0.635 and 0.891 between canine calcification, assessed by Nolla, and SMI, assessed by Fishman, for males and females, respectively [33]. The described correlations between dental and bony maturations were neither restricted to one method nor one anatomical site. Chaudhry et al. reported that dental age based on calcification of permanent teeth (using Demirjian method) significantly correlated with the ossification of carpal bones showed by radiography (*r* = 0.752). Moreover, dental and skeletal ages correlated similarly with chronological ages (*r* = 0.650, *r* = 0.620, respectively) [66]. The grade of second molar calcification and cervical vertebrae maturation exhibited a similar positive correlation (r = = 0.618) [26].

While we observed that females approached the different SMIs earlier than males, these differences were less apparent when the dental age was assessed, where the girls and boys showed very close medians of chronological ages. Fishman acknowledged the sex variations in the skeletal age, including the age of onset and the progression of bony maturation. They concluded that these variations are mostly evident around the time of the accelerated growth velocity [17]. The bone maturation process takes longer in males [9], which was agreed in previous studies [33, 69, 70]. Similarly, Brotons et al. reported that females approach the different stages of skeletal maturation earlier than males [26].

Consistently, two previous studies denied significant sex differences in dental mineralization using the Nolla method [22, 59]. Indeed, earlier dentation in females was well recognized [57]. Dental mineralization also exhibits similar sexual variances, where teeth mineralization in females precedes the males. However, the early phases of dental mineralization are very close for both sexes up to puberty, where the girls exhibit greater development [71]. Among Coroatain children, there were significant sex differences in the mineralization of the mandibular wisdom tooth [53]. It was previously recognized that there were sex variations in dental mineralization, with girls generally showing faster growth rates [72]. Holtgrave et al. mentioned that dental mineralization in boys is comparable to that in girls only until the age of 9–10 in the former. After that age, there were significant sex variations; hence, the Nolla method and other methods used in dental mineralization are more accurate in younger ages [36, 61].

The current study proved that the chronological age estimation using dental and skeletal maturation showed better results closer to the actual age among boys than girls. Likewise, Maber et al. and Miloglu et al. et al. reported better age estimation using the Nolla method among boys than girls [22, 56]. An earlier study conveyed that the Nolla method was an accurate age predictor in girls only up to late childhood, approximately ten years [23]. Inversely, Alqadi and Abuaffan concluded that the Fishman method was more precise in Yemeni girls than boys, in which the chronological and dental ages did not vary significantly among girls aged 11–14 years [31]. The skeletal age matched the chronological ages of Indian girls aged 12, 13 and 14 [32].

The present study showed better performance of the proposed models than previous studies. Alqadi and Abuaffan reported adjusted R^2^ values of 55% for females and 61% for males using the Fishman skeletal method [30]. The same authors in another recent study among Yemeni children reported R^2^ between 0.55 and 0.61 for an age prediction model based on the Fishman method and R^2^ between 0.77 and 0.79 for another model using the Nolla method, confirming the superior performance of dental age [31]. Cortés et al. established a formula for estimating the chronological age using the dental age assessed by the Nolla method. Their models yielded lower performance (adjusted R^2^ of 0.7371 in Spanish girls and 0.7850 in boys) compared to the present study [11]. Mostad et al., introduced a mathematical model to help make optimal decisions in forensic age assessment. Although they reported a weak positive conditional correlation between the maturity of the distal femur and the third molar, they advised combining sound radiological methods, statistical analysis, and presenting the results to describe the obtained findings as an age assessment model [73].

### Limitations and recommendations

Among the limitations of the current study is the negligence of multiple confounders, including the hereditary, environmental, functional, nutritional, and metabolic alterations and any present pathological conditions that might influence the process of skeletal and dental development [11, 48]. Additionally, some selection bias may result from recruiting the sample from orthodontic cases. Determining the chronological age, as per birth proofs, as an expression of an individual’s real age was an inherent limitation. In some cases, the recorded chronological age may not precisely reflect the actual age if the birth record was issued later than the day of birth. So, the documented chronological age may not accurately reflect the actual age, leading to potential discrepancies in age estimation studies. Another limitation is that assessing the age using SMIs necessitated performing additional hand and wrist radiography, which might be inaccessible, particularly in rural settings. The lack of explicit references specified for different populations reduces the untrustworthiness of using skeletal age as an absolute age predictor [74].

This study acknowledges the difference in the biological processes of teeth mineralization and bony maturation. These distinct developmental pathways may introduce variability when directly comparing ages. Therefore, while both methods are valuable, caution should be exercised when interpreting results that compare these differing biological parameters in age estimation studies. We recommend future research on validating the proposed models on multiethnic samples and suggesting specific charts applicable to different populations. Besides, we recommend including only individuals with verified birth records.

## Conclusion

The present study has shown that both the Fishman and Nolla methods were valid and reliable age predictors with variable sex and age group accuracy in Egyptians children and adolescents. However, the age estimation using the Nolla method, depending on the dental mineralization, showed superior performance compared to skeletal maturation using the Fishman method. However, both methods significantly correlated with the chronological age and each other. The models incorporating only the Nolla scores adapted to our sample were the most accurate predictors of chronological age in both sexes.

## Electronic supplementary material

Below is the link to the electronic supplementary material.

Supplementary Material 1

## Data Availability

The datasets used and/or analyzed during this study are available from the corresponding author on reasonable request.
