# Supplementary material for: Bridging gaps in age estimation: a cross-sectional comparative study of skeletal maturation using Fishman method and dental development using Nolla method among Egyptians
Source: Int J Legal Med. 2025 Jan 6;139(2):695–714. doi: 10.1007/s00414-024-03394-x (PMC11850478; doi:10.1007/s00414-024-03394-x)
Supplement: Supplementary file 1 — Supplementary Material 1 [file 414_2024_3394_MOESM1_ESM.docx]

**Supplementary Table 1** Intra-observer and inter-observer agreement tests

| **Agreement** | **Weighted Cohen’s Kappa** | **95% CI** | ***P* value** |
| --- | --- | --- | --- |
| **Intra-observer (SMI)** | 0.993 | 0.990 - 0.997 | ---- |
| **Inter-observer (SMI)** | 0.991 | 0.987 - 0.995 | ---- |
| **Agreement** | **Interclass correlation (ICC)** | **95% CI** |  |
| **Intra-observer (Nolla score)** | 0.999 | 0.999 - 0.999 | <0.001* |
| **Inter-observer (Nolla score)** | 0.999 | 0.999 - 0.999 | <0.001* |

Interpretation: For weighted Cohen’s Kappa: values above 0.90 based on 82-100% of the data indicates almost perfect agreement; For ICC: values greater than 0.90 indicate excellent reliability; *: significant at *P*<0.05
